# Apparent Digestibility of Macronutrients and Fatty Acids from Microalgae (*Schizochytrium* sp.) Fed to Rainbow Trout (*Oncorhynchus mykiss*): A Potential Candidate for Fish Oil Substitution

**DOI:** 10.3390/ani11020456

**Published:** 2021-02-09

**Authors:** Amélie Bélanger, Pallab K. Sarker, Dominique P. Bureau, Yvan Chouinard, Grant W. Vandenberg

**Affiliations:** 1 Département des Sciences Animales, Pavillon Paul-Comtois, Université Laval, Quebec, QC G1V 0A6, Canada; amelie.belanger@live.ca (A.B.); Yvan.Chouinard@fsaa.ulaval.ca (Y.C.); 2 Environmental Studies Department, University of California, Santa Cruz, CA 95064, USA; psarker@ucsc.edu; 3 Fish Nutrition Research Laboratory, Department of Animal and Poultry Science, University of Guelph, Guelph, ON N1G 2W1, Canada; dbureau@uoguelph.ca

**Keywords:** microalgae, *Schizochytrium* spp., rainbow trout, fish oil, biomass, digestibility, nutrients, lipid, fatty acid

## Abstract

**Simple Summary:**

Aquaculture is the world’s fastest growing sector of the global food system. Aquaculture feed producers are seeking substitutes for fishmeal and fish oil to develop more sustainable feeds. Marine microalgae show promise as potential ingredients in aquafeeds; however, the literature lacks data on the digestibility and availability of macronutrients and individual fatty acids for omega 3-rich microalgal biomass, *Schizochytrium* spp., in rainbow trout. This is an important gap to fill because, among marine microalgae, this species is the most frequently used in fish feed, particularly as a dietary supplement or mixed with other ingredients; digestibility data would guide more judicious inclusion of this microalga biomass. High digestibility of macronutrients, energy and fatty acids showed that *Schizochytrium* spp. is a high-quality substitute for fish oil and potential candidate as a supplement of LC-PUFA in trout feed with vegetable oils. Digestibility is influenced by temperature and several trials have shown increased macronutrient digestibility with increasing temperature. There is an urgent need to determine the effect of temperature on lipid and fatty acid digestibility. We found that the digestibility of nutrients in rainbow trout maintained at 8 °C or 15 °C was not significantly different. However, the digestibility of DHA omega 3 was significantly higher at 8 °C than at 15 °C.

**Abstract:**

Aquaculture feed formulation has recently turned its focus to reduce the reliance on marine-derived resources and utilise alternative feedstuffs, as an approach to improve the environmental sustainability of the aquaculture sector. The fish oil market is highly volatile, and availability of this commodity is continuously decreasing for use in aquaculture. Currently, a growing number of commercial efforts producing microalgae are providing omega 3-rich oil for sustainable aquaculture feed. This study was focused to determine the nutrient digestibility of a marine microalga, *Schizochytrium* spp., which is rich in docosahexaenoic acid (DHA) and long-chain polyunsaturated fatty acids (LC-PUFA), as a novel dietary lipid source that could be utilized effectively by rainbow trout (*Oncorhynchus mykiss*). A whole-cell *Schizochytrium* spp. biomass was used in the digestibility experiment at two different temperatures, 8 °C and 15 °C. No significant differences were detected between the two temperatures for the apparent digestibility coefficients (ADCs) of the dry matter (94.3 ± 4.9%), total lipids (85.8 ± 0.0%), crude proteins (89.5 ± 1.8%), energy (83.1 ± 1.7%) and fatty acids (85.8 ± 7.5%). The ADCs of the nutrients, energy, DHA and other fatty acids showed that *Schizochytrium* spp. is a high-quality candidate for fish oil substitution and supplement of LC-PUFA in fish feed with vegetable oils.

## 1. Introduction

Aquaculture is an extremely diverse industry, which is expanding rapidly. Consumer demand for farmed fish is growing while the amount of fish from capture fisheries for human consumption is static or declining. Aquaculture now accounts for more than 50% of seafood for human consumption [1,2,3,4]. To meet the demand of aquaculture, high-energy omega 3-rich ingredients are required to produce sustainable aquaculture feed. However, a steady supply of fish oil and fishmeal, major ingredients of aquaculture feeds, is becoming a major concern due to the overexploitation of wild marine forage fish [5,6,7]. For example, current aquaculture feeds contain terrestrial crop oils to partly substitute fish oil, due to their vast availability and low cost [8]. Several studies have demonstrated that a significant portion of fish oil can be replaced with vegetable oils in the feeds of salmonids, without compromising survival, growth and feed efficiency [9,10,11]. However, studies showed that substitution of vegetable oils in aquafeeds influences the fillet fatty acid profile in fish. High levels of fish oil substitution can significantly compromise the concentration of omega 3 long-chain polyunsaturated fatty acids (LC-PUFA), such as docosahexaenoic acid (DHA) and eicosapentaenoic acid (EPA), in farmed salmonids, well recognized for their beneficial cardiovascular and cognitive properties to humans who consume the fish [3,4,12,13,14,15]

*Schizochytrium* spp. is a marine microalga that contains 18–22% DHA by dry weight (Advanced BioNutrition, Columbia, MD, USA). Inclusion of this ingredient in terrestrial oil-based aquaculture feeds could maintain the levels of DHA in the flesh of farmed salmonids. This study aimed to determine the digestibility of *Schizochytrium* spp. when added to regular rainbow trout (*Oncorhynchus mykiss*) feed. This is the first report where we determined the macronutrients and fatty acid digestibility of a *Schizochytrium* species’ whole cells in rainbow trout. It is generally assumed that digestibility is influenced by temperature and a number of studies have shown increased macronutrient digestibility with increasing water temperature [16,17], even though there are also reports showing no significant effect on lipid and fatty acid digestibility [18]. On the contrary, a few studies have shown that decreased temperatures will increase the fecal holding time and thereby increase digestive capacity [19,20]. Therefore, the nutrient digestibility experiment was conducted at two water temperatures (8 °C and 15 °C) to determine the changes in the *Schizochytrium* species’ apparent digestibility coefficients when rainbow trout are exposed to the minimum and maximum temperature.

## 2. Materials and Methods

All experimental procedures were approved by the Comité de Protection des Animaux de l’Université Laval (animal care/ethics committee).

### 2.1. Feed Design and Formulation

The chemical composition of the *Schizochytrium* spp. (Thraustochytriaceae family; Advanced BioNutrition, Columbia, MD) is reported in Table 1. To investigate the digestibility of whole cells of *Schizochytrium* spp., two feeds were formulated: one is a nutritionally complete reference feed based on the requirements for rainbow trout [4,21], and an algae diet (test diet) with 30% of *Schizochytrium* spp. replacing the reference feed [22] (Table 2). The reference diet was combined with the test microalga species (dried whole cells of *Schizochytrium* spp.) at a 7:3 ratio to produce an algae diet to determine the nutrient digestibility by following the standard apparent digestibility protocol described in our previous studies with rainbow trout and tilapia [4,22,23,24]. The fatty acid profile and chemical composition of the diets are presented in Table 3. Sipernat 50^TM^ (acid-insoluble ash) from Degussa AG, Frankfurt, Germany, was included in the diet as an indigestible marker to determine the apparent digestibility coefficients (ADCs) for the fatty acids and other macronutrients (protein, lipid, and energy). The feed pellets (4 mm die) were produced with a California Pellet Mill (model CPM CL-5, California Laboratory Pellet Mill Co., Crawfordsville, IN, USA), dried under forced air at room temperature for 24 h, and finally sieved before use.

### 2.2. Fish and Feeding

Prior to the digestibility experiment, rainbow trout (146.4 ± 4.1 g) were randomly allocated in twelve 60-L rectangular tanks (2 diets × 2 temperatures × 3 replicates = 12 tanks) in a freshwater recirculating system at the Laboratoire de Recherche des Sciences Aquatiques (LARSA) of Université Laval (Quebec, QC, Canada). All tanks were stocked with 17 fish to reach approximately 42 kg/m^3^ biomass per tank. The experiment was designed to collect feces from three replicate tanks per treatment (three fecal samples per treatment; *n* = 3), and the reference diet and algae diet (*Schizochytrium* spp.) were randomly assigned to the twelve tanks. The digestibility experiment was conducted at two water temperatures, at 8 ± 1 °C and at 15 ± 1 °C. The environmental conditions of the experiment were maintained within the limits recommended for rainbow trout by the National Research Council and our recent digestibility experiment [4,21,25].

Prior to the commencement of the experiment, trout were acclimated three days to the feed and temperatures. The fish were carefully hand-fed at apparent satiation twice daily at 9:00 a.m. and 4:00 p.m. Feces were collected twice daily before each meal for nine days using a modified Guelph system based on the method previously described [22]. After each collection, feces were stored at −20 °C. Finally, fecal samples were thawed in a refrigerator at 4 °C, centrifuged to remove excess water and freeze-dried for chemical analysis to determine the apparent digestibility coefficients (ADCs) according to the method previously described [22]:

a = 1, ADC = 1 − (F/D × D_i_/F_i_)

where D = % nutrients (or MJ g^−1^ gross energy) of the diet; F = % nutrients (or MJ g^−1^ gross energy) of the feces; D_i_ = % digestion indicator (acid insoluble ashes; AIA) of the diet; and F_i_ = % digestion indicator (AIA) of the feces.

The apparent nutrient digestibility of the *Schizochytrium* spp. was determined according to the method previously described [26], and subsequently the equation has been reviewed, critiqued, modified and described [23,25]:

ADC_algae_ ingredient = ADC_algae diet_ + ((ADC_algae diet_ – ADC_ref. diet_) × (0.7 × D_ref_/0.3 × D_algae_ )), where D_ref_ is the percentage of nutrients or MJ g^−1^ gross energy of the reference diet; and D_algae_ is the percentage of nutrients or MJ g^−1^ gross energy of the algae diet.

### 2.3. Chemical Composition

The reference diet, *Schizochytrium* spp. algae diet, whole cells of *Schizochytrium* spp. and feces were analyzed for dry matter, ash, crude protein, total lipid, gross energy and fatty acid profile to quantify ADCs. Samples were dried in a forced air oven at 105 °C overnight for dry matter analysis. Samples were weighed (before and after drying), followed by cooling in a desiccator [27]. The ash content of the samples were achieved by dry ashing in porcelain crucibles in a muffle furnace at 500 °C overnight and reported in dry weight [27]. Gross energy was analyzed, and estimated as a as MJ/kg DM using bomb calorimetry (Parr Instrument Company Inc., Moline, IL, USA). LECO (model FP-2000; LECO Corporation, St-Joseph, MI, USA) was used to measure the crude protein content of the samples and the nitrogen (N) conversion factor of N × 6.25, expressed as dry weight.

The total lipid content was analysed via a Soxhlet HT-TECATOR^®^ extractor (Soxtec System HT12, Foss Tecator AB; Hoganas, Sweden), and diethyl ether was used as solvent at 100 °C. Before the extraction, 1% of British Thermal Unit (BHT) was added to eliminate oxidation of the fatty acids, as the samples were further prepared to assess the fatty acid profiles. Total lipids were reported as dry weight. Samples were blown dry under nitrogen and, prior to the lipid extraction and fatty acid analysis, acid hydrolysis was performed for the fecal samples [28,29]. The AOAC FAME procedure [30] was followed to determine the fatty acid content of the samples. Fatty acid samples were analyzed via a GC FID gas chromatograph from Hewlett-Packard, model 5890 series II (Palo Alto, CA, USA), equipped with a CP-Sil 88 capillary column (100 m × 0.25 mm) with helium carrier gas. Oven temperature was controlled at 80 °C for 1 min before being raised to 215 °C (2 °C min^−1^) for 30 min and maintained for 98 min. The injector temperature was maintained at 220 °C and the detector temperature was maintained at 230 °C. Pure methyl ester standards (Nu Chek Prep; Elysian, MN, USA) were used to identify and quantify the fatty acid peaks. Individual components were identified by comparing the retention times with those of the standards and quantified by calculating the area under the curve with the ChemStation Rev: A 10.01 program (Agilent Technologies; Santa Clara, CA, USA). The fatty acid profiles of the fish oil and *Schizochytrium* spp. were reported previously [31]. In addition, we analyzed Sipernat 50^TM^, the acid-insoluble ash (AIA) in the feed and feces according to the standard method [32].

### 2.4. Statistical Analysis

One-way analysis of variance (ANOVA) was applied for determining any significant differences in the apparent digestibility coefficients for macronutrients and fatty acids in the reference and *Schizochytrium* spp. algae diet (test diet), as well as for temperatures. Before the ANOVA, the digestibility percentages values were arcsin transformed and normality was assessed via a Kolmogorov–Smirnov test. The digestibility fatty acid values were tested for a normal distribution, and when significant differences were found, we compared the treatment means using Tukey’s test of multiple comparisons, with a 95% confidence interval (probability value of *p* < 0.05). The data are presented as the mean ± standard deviation. All statistical analyses were carried out using SAS 8.0 (SAS Institute Inc., Cary, NC, USA).

## 3. Results

No significant differences were found between temperatures for the ADCs of dry matter (overall mean, 94.3%), total lipid (85.8%), crude protein (89.5%) and energy (83.1%) for *Schizochytrium* spp. (Table 4).

The ADCs of most of the major fatty acid and fractions, except for 22:6*n*-3 DHA, were not significantly different among temperatures. The ADCs of the saturated fatty acids (SFA; 77.4% and 70.6% for 8 and 15 °C, respectively) were not significantly different (Table 5). The mean ADC (8 and 15 °C) for each fraction of SFA was of 76.0% for 14:0, 69.3% for 16:0 and 57.0% for 18:0. We did not detect any significant differences for total MUFA (90.4%) and its fraction for 16:1 (92.9%), 18:1*n*-9 (88.2%) and 24:1 (69.3%).

The ADC of 22:5*n*-6 (total *n*-6) was not significantly different between the two temperatures, and the mean value was 98.7%. The ADC of the total *n*-3 fatty acids (98.3%) did not differ significantly. No significant differences were detected between two temperatures for the ADCs of the 18:3*n*-3 fatty acid (82.2%) and 20:5*n*-3 (98.6%). However, 22:6*n*-3 was detected significantly higher at 8 °C than at 15 °C (*p* < 0.05). Furthermore, the ADC for total polyunsaturated fatty acids (PUFA) (98.5%) did not show a significant difference between temperatures.

## 4. Discussion

This study aimed to examine the digestibility of DHA-rich *Schizochytrium* spp., a marine microalga, at two different temperatures, to evaluate the feasibility of using this microalga in aquafeed for rainbow trout. The results of this study showed that the overall nutrient digestibility in rainbow trout did not vary between two temperatures, 8 °C and 15 °C. Similarly, it has been reported that there is no significant difference in the dry matter, protein, lipid and energy digestibility in trout rearing at 7 °C, 11 °C and 15 °C [33]. A recent study conducted with rainbow trout revealed that increasing the water temperature (up to 15 °C) did not cause an increase in lipid and fatty acid digestibility; however, the elevated water temperature of 20 °C tended to cause a reduction in the digestibility of most SFA, MUFA, PUFA and total lipids when compared with 15 °C water temperature [34]. It has been demonstrated that digestibility did not differ between 9 °C and 15 °C in rainbow trout (37 to 113 g) [35]. However, the ADC was slightly higher at 18 °C, because the basal metabolic rate was increased at this temperature [36]. It was reported that Atlantic salmon kept at very low temperature (3 °C) exhibited a significantly reduced digestibility, leading to increases in fecal wax esters and triacylglycerols [37].

It is generally assumed that higher temperature increases metabolism, including higher enzyme activities, with an increased amount of lipase in the midgut of fish, and faster absorption [38,39]. Our digestibility results at 15 °C are not in agreement with a prior study but the digestibility at lower temperatures was similar [35]. The apparent digestibility of lipid and fatty acids was reported higher at higher temperature when trout fed on lard and tallow [36]. However, similar to our results, the ADCs of the lipid and fatty acids were not significantly different for rapeseed, soybean, and linseed oils among temperatures. The authors concluded that lard and tallow should therefore be avoided as a source of lipid in diets for cold-water fish [36].

With the exception of 22:6*n*-3 fatty acid, other major fatty acids and their fractions (SFA, MUFA, PUFA, *n*-3, and *n*-6) were not influenced by water temperature (Table 5). It has been reported that a decrease in temperature, from 15 °C to 7 °C, did not significantly reduce the total MUFA and PUFA apparent digestibility [40].

In recent years, marine microalgae have gained considerable attention as possible alternative oil sources for aquaculture feed mainly due to the LC-PUFA; for example, marine microalgae like *Schizochytrium* spp. have high contents of LC-PUFA (especially rich in DHA content) [2,3,4,31,41]. The results of our study showed that the lipid and fatty acids contents in *Schizochytrium* spp. are highly digestible for rainbow trout (Table 4 and Table 5). The average (8 °C and 15 °C) dry matter digestibility (94.3 ± 7.8%) of *Schizochytrium* spp. was high as the digestibility (95.0 ± 0.3%) of *Calanus finmarchicus* in Atlantic salmon (*Salmo salar*) of 500 g held at 10 °C in seawater [42] (Table 4). The protein digestibility results herein are also in agreement with the previous studies and varies from 75 to 95% [21,25]. An ADC of 92% for crude protein [36], and an ADC between 92 and 95% for 37 to 113 g rainbow trout have been reported [33]. However, other studies reported a lower value of 84.7% for the ADC of protein for Atlantic salmon [43]. The whole cells of *Schizochytrium* spp. has also a high digestibility for gross energy, with a mean (8 °C and 15 °C) of 83.1 ± 2.7%, which is similar (82.5%) to those values obtained for Atlantic salmon [44]. It has been reported that the gross energy digestibility of animal by-products varies between 68 and 99% in rainbow trout [43]. Our results of the lipid ADC were in the range of 80% to 93%, in agreement other studies [4,22,35,36] (Table 4). The apparent digestibility of lipid was between 79.2 and 92.5% in rainbow trout (initial weight 250 g) raised at 11.9 ± 0.4 °C [10].

Previous work reports the apparent digestibility of the SFA of microalgae varies in the range between 60 and 99%, and our results fall within this range [10,34,42] (Table 5). The ADC of SFA was similar to the ranges when Atlantic salmon were fed varying levels of n-3 and SFA [45]. It was reported that the ADCs for total MUFA were between 90.2 and 95.2% [10], and other researchers reported between 93.5 and 96.0% [42]. The digestibility of total MUFA in our study at 8 °C is slightly lower (87.4 ± 4.8%), as reported by these authors [39]. The ADC at 15 °C is similar to the one observed in a feeding trial conducted with rainbow trout fed palm fatty acid distillate, and reported ranges between 95.3 and 95.7% at 15 °C water temperature [34]. The digestibility of total *n*-3 and *n*-6 fatty acids was detected extremely high, close to 100%. These results are in line with the previous studies, and the range was between 93 and 99% [10,34,42]. In general, the apparent digestibility of the fatty acids decreases with increasing chain length, but increases with increasing unsaturation [34,46,47]. According to Sigurgisladottir et al. (1992) and Turchini et al. (2009), the ADC of an individual fatty acid varies due to its melting point, and when the melting point is higher, the digestibility can be lower [13,46]. Preferential absorption of PUFA, compared to the MUFA and SFA of rainbow trout, is in line with the fatty acid digestibility in rainbow trout and Atlantic salmon reported by other investigators [10,24,34,40,47]. As mentioned above, the digestibility of a fatty acid is dependent on its melting point: the lower the melting point (corresponding to a higher unsaturation degree like DHA), the higher the apparent digestibility [48]. A change in water temperature highly impacts the digestibility through its influence on lipid fluidity, the effect of a temperature increase being more pronounced for the fatty acids with higher melting points [49]. Although we did not measure the lipase activity in this study, the reduction in DHA digestibility at higher temperature in this study could be explained by a reduced lipase activity and/or an increased intestinal transit rate, reducing the contact time between the lipases and dietary fatty acids [34]. However, the results suggest more research is needed to understand the mechanisms behind the temperature-related differences in digestibility.

It has been reported that an increased water temperature of 5 °C reduced the apparent digestibility of MUFA, n-6 PUFA (particularly LA), and n-3 PUFA (particularly EPA and DHA) in rainbow trout fed a palm fatty acid distillate-based diet (FFA-rich oil), whereas the apparent digestibility of SFA was not affected [34]. A reduced lipase activity has also been reported for marine species, yellowtail kingfish (*Seriola lalandi*), when the rearing temperature was increased from 24 to 27 °C [50]. Furthermore, it is important to note that the effect of water temperature on fatty acid digestibility may be dependent on the dietary lipid source. More research is thus required on the digestibility of sustainable lipid source alternatives in aquaculture feeds.

## 5. Conclusions

The inclusion of microalgae in fish diets is still in an early stage of development for the aquafeed industry. Understanding the digestibility of ingredients is essential to formulate sustainable aquafeeds, but data on nutrient digestibility for novel feed ingredients like microalgae in aquafeeds is largely unavailable. These constraints may result in the inaccurate formulation of economically viable and environmentally sustainable feed formulations [4]. This study was focused on determining the digestibility of DHA-rich *Schizochytrium* spp., a marine microalga, at two different temperatures (8 °C and 15 °C), to evaluate the feasibility of using this microalgal species in aquafeed for rainbow trout aquaculture. Overall, this study suggested that the digestibility of nutrients in rainbow trout maintained at 8 °C or 15 °C were not significantly different. The digestibility of *Schizochytrium* spp. for lipid, fatty acids, and DHA shows that this marine microalgal species is a high-quality candidate for a potential substitute to fish oil and DHA supplementation in a trout diet formulation. As marine microalgae are considered among the most promising sustainable ingredients, future research should be warranted to blend this LC-PUFA-rich microalgal species with other protein-rich microalgae and terrestrial crop oils to fully replace wild-caught fishmeal and fish oil in the diet of salmonids. It is key to measure the effects of different levels of fishmeal and fish oil replacements with microalgae blends on the fish growth, feed efficiency, and flesh nutritional quality of fish, including DHA deposition. In the future, the environmental impacts and economic potential of microalgae in aquaculture feeds also should be fully evaluated.

## Figures and Tables

**Table 1:** Chemical composition of the *Schizochytrium* sp. used in this experiment ^1^.

Chemical Composition	g kg^−1^ DM ^2^
Crude protein	150
Total lipid	417
Fiber	10
Ash	100
Gross energy (MJ kg^−1^ DM)	24.33

^1^*Schizochytrium* spp. was used as the algal biomass (Advanced BioNutrition, Columbia, MD, USA); ^2^ Dry matter (940 g kg^−1^ DM).

**Table 2:** Ingredient composition (g kg^−1^) of the experimental diets.

Ingredient	Feed	
	Reference Diet	Algae Diet
Fish meal	300	210
Corn gluten meal	170	119
Wheat middlings	163	114
Soybean meal	130	91
Whey	100	70
Vitamin/mineral premix ^1^	5	4
Fish oil, herring	112	78
Sipernat 50^TM 2^	20	14
*Schizochytrium* spp. Biomass ^3^	0	300

^1^ Salmonid vitamin/trace mineral premix from Corey (Fredericton, NB). ^2^ Sipernat 50^TM^ (Degussa AG, Frankfurt, Germany). ^3^ Advanced BioNutrition, Columbia, MD.

**Table 3:** Chemical composition and fatty acid profile of the feeds.

Chemical Composition	Feed	
	Reference Diet	Algae Diet
DM (%, as fed basis)	93.4	95.6
Crude protein (% of DM)	42.0	34.6
Total lipid (% of DM)	17.7	27.0
Ash (% of dry matter)	10.0	9.7
Gross energy (MJkg^−1^ DM)	22.7	24.1
Fatty acid (% total fatty acids)		
14:0	6.7	7.4
16:0	21.8	23.3
18:0	4.0	2.0
Total SFA ^1^	40.9	36.8
16:1	6.0	2.6
18:1*n*−9	11.9	4.9
24:1	0.5	0.3
Total MUFA ^2^	22.8	9.2
22:5*n*−6	2.4	11.1
Total *n*−6	2.4	11.1
18:3*n*−3	2.5	1.1
20:5*n*−3	12.3	6.8
22:6*n*−3	17.5	33.7
Total *n*−3	32.6	41.9
Total PUFA ^3^	35.0	53.0
*n*−3/*n*−6	13.4	3.8

^1^ SFA, saturated fatty acid includes 15:0, 17:0, 20:0, 22:0 and 24:0. ^2^ MUFA, monounsaturated fatty acid. ^3^ PUFA, polyunsaturated fatty acid.

**Table 4:** Apparent digestibility coefficients (%, mean ± standard error, *n* = 3) of the macronutrients and gross energy in the *Schizochytrium* spp. ingredient for rainbow trout at two water temperatures, 8 °C and 15 °C.

Macronutrient	Water Temperature	
	8 °C	15 °C
Dry matter	97.8 ± 1.2	90.8 ± 11.5
Crude protein	88.2 ± 1.2	90.8 ± 0.7
Total lipid	85.8 ± 5.1	85.9 ± 4.4
Gross energy	81.9 ± 2.6	84.3 ± 3.1

Data represent the mean ± standard deviation of three replicate tanks (measured duplicate fecal samples from each tank).

**Table 5:** Apparent digestibility coefficients (%, mean ± standard error, *n* = 3) of the fatty acids in rainbow trout fed *Schizochytrium* spp. when replacing 30% of a reference diet at two water temperatures, 8 °C and 15 °C.

Fatty Acids	Water Temperature	
	8 °C	15 °C
14:0	77.0 ± 11.6	75.6 ± 2.7
16:0	70.8 ± 10.6	68.7 ± 3.6
18:0	49.9 ± 11.0	64.8 ± 3.7
Total SFA ^1^	77.4 ± 8.8	70.6 ± 3.4
16:1	90.7 ± 3.3	95.3 ± 0.2
18:1*n*−9	84.4 ± 6.7	92.0 ± 0.6
24:1	63.6 ± 14.4	75.4 ± 3.8
Total MUFA ^2^	87.5 ± 5.2	92.1 ± 0.5
22:5*n*−6	99.3 ± 0.3	98.2 ± 0.1
Total *n*−6	99.3 ± 0.3	98.2 ± 0.1
18:3*n*−3	75.8 ± 12.8	88.7 ± 1.3
20:5*n*−3	98.4 ± 1.2	98.7 ± 0.1
22:6*n*−3	99.1 ± 0.1 ^b^	98.5 ± 0.1 ^a^
Total *n*−3	98.4 ± 0.7	94.9 ± 0.1
Total PUFA ^3^	98.7 ± 0.5	98.5 ± 0.1

Data represent the mean ± standard deviation of three replicate tanks (measured duplicate fecal samples from each tank). ^a,b^ Values with different superscripts are significantly different (*p* < 0.05). ^1^ SFA, saturated fatty acid, which includes 15:0, 17:0, 20:0, 22:0 and 24:0. ^2^ MUFA, monounsaturated fatty acid. ^3^ PUFA, polyunsaturated fatty acid.

## Data Availability

We have presented extensive data generated by this study in the tables. Contact the corresponding author for further requests.
